# Whole-Genome Sequence of the Phlox Powdery Mildew Pathogen Golovinomyces magnicellulatus Strain FPH2017-1

**DOI:** 10.1128/MRA.00852-19

**Published:** 2019-09-05

**Authors:** Coralie Farinas, Emile Gluck-Thaler, Jason C. Slot, Francesca Peduto Hand

**Affiliations:** a Department of Plant Pathology, The Ohio State University, Columbus, Ohio, USA; Vanderbilt University

## Abstract

Powdery mildew (PM) fungi are obligate biotrophs capable of infecting diverse plant hosts, ranging from monocotyledonous agricultural crops to dicotyledonous ornamental crops. The PM lifestyle poses significant challenges for studying these pathogens in isolation from their host. We present a draft genome of Golovinomyces magnicellulatus, a host-specific PM on *Phlox* species.

## ANNOUNCEMENT

Golovinomyces magnicellulatus (Leotiomycetes, Ascomycota) is an obligate host-specific fungal biotroph that causes powdery mildew (PM) disease on ornamental plants in the *Phlox* genus (1). Due to difficulties in growing PM fungi under axenic conditions, little is known regarding the genetic and evolutionary bases of their lifestyles, presenting an opportunity to gain insight through a genome-focused approach.

*G. magnicellulatus* strain FPH2017-1 was isolated from Phlox paniculata in Leipsic, Ohio. A single spore was isolated on a detached leaf bioassay (2) and grown on *P. paniculata* ‘Starfire’ plants in a growth chamber. Spores were harvested periodically over 1 month by rinsing infected leaves with 0.1% Tween solution and then filtering with Miracloth and stabilizing using 10 mM Tris buffer (pH 7). The solution was centrifuged, and the resulting pellet was immersed in liquid nitrogen and kept at –80°C. DNA was extracted from the pellet using the DNeasy plant minikit (Qiagen).

DNA libraries were prepared using the NEBNext Ultra II DNA library prep kit and sequenced using the Illumina MiSeq PE300 platform. Unsheared DNA extracts were prepared using a ligation sequencing kit (SQK-LSK109) and sequenced using MinION (Oxford Nanopore Technologies).

Illumina sequencing generated 17,742,739 reads (35 to 300 bp long) at 46× coverage (Table 1). Reads were trimmed using Trimmomatic v.0.36 (3) with the options ILLUMINACLIP, TruSeq3-PE.fa:2:30:10, CROP:290, SLIDINGWINDOW:10:25, HEADCROP:10, and MINLEN:100 (4). Nanopore sequencing generated 427,831 reads (46 to 42,472 bp long) at 4× coverage (Table 1). The reads were quality filtered using Albacore v.2.3.1 (5). Iterative BLASTn searches against the NCBI nucleotide database (last accessed 11 February 2019) in conjunction with BBSplit v.37.93 (6) were used to identify and remove contaminant reads that had >75% identity and >50% query coverage to database entries originating from nonfungal organisms. We then performed *de novo* hybrid genome assembly using SPAdes v.3.12.0 (7) and identified known and *de novo* repeat elements using RepeatModeler v.1.0.11 (8).

**TABLE 1:** Summary statistics of assembly and annotation of *G. magnicellulatus*

Parameter	Value
Assembly	
Genome size (Mb)	129.9
Avg coverage (×) (no. of Illumina reads)^*a*^	46 (97)
Avg coverage (×) (no. of Nanopore reads)^*a*^	4 (60)
No. of contigs	84,604
*N*_50_ (bp)	4,118
Longest scaffold (kbp)	197
GC content (%)	44
BUSCO^*b*^ (% recovered)	88.2
Annotation	
Total no. of protein-coding genes	8,172
Avg gene length (bp)	1,764
No. of coding sequences^*c*^	8
No. of repeat sequences^*c*^	40
No. of proteins with at least one Pfam domain^*d*^	6,396
No. of secreted proteins^*e*^	304

a Percentage of reference bases covered, estimated using BBMap v.37.93 (6).

b Sordariomyceta data set.

c Percent assembly size.

d Identified using InterProScan v.5.25-64 (15).

e Identified using SignalP-5.0 (16) and TMHMM v.2.0 (http://www.cbs.dtu.dk/services/TMHMM/) to exclude transmembrane proteins.

We annotated the assembly using three iterations of a MAKER v.2.31.9 (9) pipeline. In the first iteration, we provided MAKER with transcriptome sequencing (RNA-seq) data of Golovinomyces cichoracearum (http://genome.jgi.doe.gov/Golci1) and 10 protein data sets from other Leotiomycetes species (Blumeria graminis f. sp. hordei DH14 [http://mycocosm.jgi.doe.gov/Blugr1], *B. graminis* f. sp. hordei Race1 [https://mycocosm.jgi.doe.gov/BlugrR1_1], *B. graminis* f. sp. tritici 96224 [https://mycocosm.jgi.doe.gov/Blugra1], Erysiphe necator [https://mycocosm.jgi.doe.gov/Erynec1], *G. cichoracearum* [https://mycocosm.jgi.doe.gov/Golci1], Amorphotheca resinae [https://mycocosm.jgi.doe.gov/Amore1], Meliniomyces variabilis [https://mycocosm.jgi.doe.gov/Melva1], Sclerotinia sclerotiorum [https://mycocosm.jgi.doe.gov/Sclsc1], Rhizoscyphus ericae [https://mycocosm.jgi.doe.gov/Rhier1/], and Botrytis cinerea [https://mycocosm.jgi.doe.gov/Botci1]). For the second iteration, we provided MAKER with the *ab initio* gene predictors SNAP v.2013-02-16 (10) (trained using high-quality predictions from round 1) and AUGUSTUS v.3.3 (11) (trained using BUSCO v.3.0.1 [12]). For the final iteration, we provided MAKER with updated evidence from SNAP and AUGUSTUS (both retrained using high-quality predictions from round 2) and set the option keep_preds to 1.

Many PM genomes are estimated to range in size from 120 to 220 Mb (13), due in part to high repeat content. Conversely, PMs generally possess fewer protein-coding genes (6,000 to 7,000) than other fungi (13, 14). Our genome falls within the reported PM genome size, 129.9 Mb, while our annotation process recovered 8,172 protein-coding genes, more than generally reported (Table 1), which we attribute to the multiple lines of *ab initio* evidence used in the annotation process.

### Data availability.

This whole-genome shotgun project has been deposited at DDBJ/ENA/GenBank under the accession numbers VCMJ00000000 and PRJNA540711 (SRA database).
